# *Origanum majorana* L. Essential Oil-Coated Paper Acts as an Antimicrobial and Antioxidant Agent against Meat Spoilage

**DOI:** 10.1021/acsomega.2c00237

**Published:** 2022-03-02

**Authors:** Sulhattin Yasar, Nizam Mustafa Nizamlıoğlu, Mehmet Onurhan Gücüş, Ahsen Ezel Bildik Dal, Kübra Akgül

**Affiliations:** † Department of Food Engineering, Faculty of Engineering, Karamanoglu Mehmetbey University, Karaman 70200, Turkey; ‡ Department of Forest Products and Chemistry, Forest Industry Engineering, Faculty of Forestry, Istanbul University-Cerrahpasa, Istanbul 34320, Turkey

## Abstract

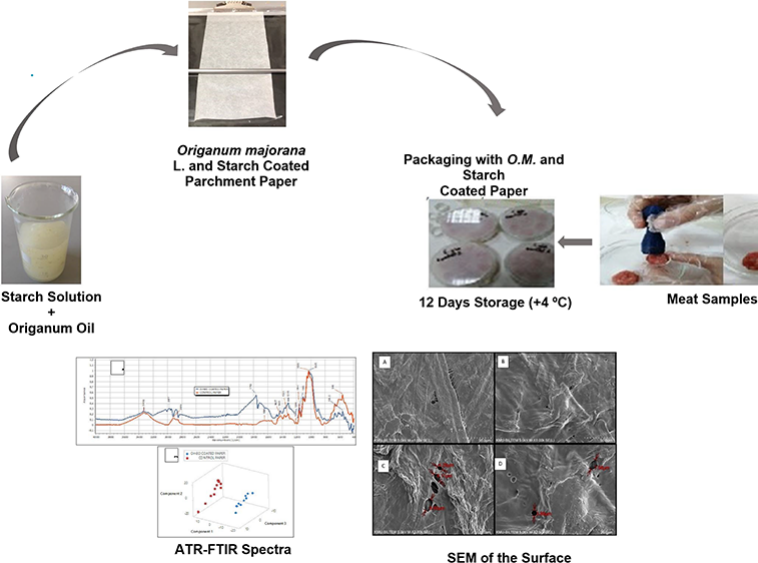

This study first-ever tested the impact of active packaging paper coated with cationic starch containing *Origanum majorana* L. essential oil with 69.26% carvacrol polyphenol on the physical, chemical, and microbiological quality of minced beef stored at +4 °C for 0, 6, and 12 days. An analysis of electron scanning microscopy and infrared spectroscopy showed origanum oil entrapment on paper. Meat samples packaged without origanum oil at 6th and 12th days of storage were unfit for consumption. In contrary, origanum oil significantly reduced microbial counts by 2.5 log 10 CFU/g, the peroxide value by 22%, lipid oxidation by 22, the pH-dependent meat spoilage value by 27%, dry matter losses by 7%, and antioxidant activity losses by 40% and restored color and odor reductions. Origanum oil extended the shelf-life of minced beef up to the 6th day of cold storage with no negative effect on meat color and odor.

## Introduction

During refrigeration of processed meat products, microbial and biochemical reactions occur to a lower or higher degree depending on storage and packaging conditions. Microbial spoilage, lipid oxidation, and color changes negatively affect the product quality and safety and reduce the shelf-life.^1^ Meat and meat products have long been treated with antimicrobial agents during their shelf-life. Of these, plant essential oils (EO) containing secondary metabolites/active agents (SM/AG) exhibit remarkable antimicrobial and antioxidant actions against meat spoilage and deterioration.^2^ It has been shown that SM/AG are responsible for beneficial effects, and they are generally regarded as safe (GRAS).^1,3^ Cinnamon essential oil has been proposed as a new active paper package. A 6% (w/w) of the cinnamon essential oil completely inhibited the growth of *Rhizopus stolonifer*, whereas a 4% of cinnamon essential oil still had strong antimicrobial activity.^3^

*Origanum majorana* L. (sweet marjoram) is an aromatic, perennial, herbaceous plant that belongs to the Lamiaceae family and the genus *Origanum* and is indigenous to Turkey and Cyprus.^4^*Origanum majorana* L. essential oil (OmEO) has been reported to have very strong *in vitro* antimicrobial and antioxidant effects due to its high carvacrol polyphenol content.^4^ However, there were almost no studies determining antimicrobial and antioxidant effects of OmEO in food packaging applications, especially in meat and meat products. In an early study, OmEO mixed with fresh sausage has been demonstrated to have a bactericidal effect, while its high dose altered the taste of sausage due to its strong odor.^5^

Of EO, thyme essential oil (TEO) is so far the most studied EO used as an antimicrobial agent incorporated with meat.^6−11^ Moreover, a synergetic antimicrobial effect of EO mixtures on chicken meat has been reported.^12^ Highly volatile aroma compounds from EO caused undesirable odor when directly mixed with the products whose shelf-life markedly extended.^7,13^ However, there is no conclusive evidence showing that EO with strong odor directly mixed with meat is confidently acceptable by the consumer.

Nanoemulsified or nano/microencapsulated films and coatings are also known as good carriers for EO to exert their beneficial effects.^9−11,14^ An active packaging film or paper produced by using OmEO has been rarely studied with meat products. A recent study showed that the active packaging films produced from pectin loaded with OmEO have excellent physical properties and increased antioxidant capacity.^15^ Similarly, Chaudhari et al.^16^ showed that the nanoencapsulation of chitosan with OmEO exhibits strong antifungal and antioxidant activity and caused *in situ* inhibition of lipid peroxidation only in plant materials. TEO alone or mixed with other EO used in edible coating materials of sodium alginate and chitosan successfully prevented meat spoilage.^16−22^ In these applications, EO has a direct contact with the products, an appreciable amount of SM/AG depending on dosing levels of EO is most likely diffused into meat, and a strong odor inevitably occurs. Although a high consumer acceptability of meat coated with TEO is reported,^23^ there is still an uncertainty whether they are most likely acceptable by consumers.^22^

A method of antioxidant active packaging with EO has been developed to overcome meat spoilage, and in such cases, the meat has no direct contact with the substances. SM/AG with antimicrobial or antioxidant properties are entrapped into packaging material and only interact with the headspace.^24−27^ In this way, it is less likely that EO may negatively modify the organoleptic properties of meat. Moreover, new research is needed to conduct trials with active packaging containing EO on prolongation of meat shelf-life due to the unavailability of data regarding the use of OmEO. Therefore, this study is a first-ever study to prevent a direct contact of OmEO with meat by using active packaging since OmEO has a strong odor.

Several technologies are used in the production of active meat packaging. These are namely film production, casting, extrusion, and coating.^28^ Of these methods, the paper coating with EO is a simplified and low-cost technology. To our best knowledge, there is no study testing the effects of active packaging paper coated with OmEO, which is emulsified in cationic starch, on the shell-life of minced beef. Recently, packaging mango fruit with a starchy film containing TEO has a strong inhibitory effect against fungal development.^29^ A nanofiber produced from eugenol EO with cationic starch also protected beef meat against bacterial deterioration up to 5 days.^30^ It was earlier well demonstrated that carvacrol polyphenol is highly soluble in oily materials and therefore can be used as a strong antioxidant in active food packaging formulation. We conducted a preliminary test, showing that a OmEO-coated oily paper may be effective to prolong the shelf-life of minced beef up to the 4th day of cold storage. Having considered all the above results, this study is first-ever conducted to test the efficacy of active paper coated with OmEO emulsified in cationic starch to prevent microbial spoilage and reduce lipid oxidation of minced beef up to 12 days of refrigerating condition.

## Materials and Methods

### Materials

OmEO was purchased from a commercial company in Antalya of the Mediterranean region of Turkey. All the chemicals used in this study were purchased from Sigma-Aldrich Chemie Gmbh, Steinheim, Germany. A cationic starch ether sample was purchased from Emstald-Starke GmbH (4459 Emlichheim). Parchment paper was selected as a base paper sample.

### Analysis of Chemical Composition of OmEO

A gas chromatography–mass spectrometric method^31,32^ was employed to analyze the chemical composition of OmEO. A device (QP-2010 Ultra, Shimadzu, Tokyo, Japan) was equipped with an HP-5MS capillary column (30 m in length, 0.25 mm in internal diameter, and 0.25 μm in thickness). One microliter of sample was injected. The injector temperature was 270 °C and the velocity of the helium carrier was 1.5 mL/min. The component of OmEO was identified by comparing their mass spectra with data records in NIST 14 and expressed as a relative percentage calculated from the normalized peak area.

### Preparation of Coating Solution and Surface Application

Paper used for coating was a plain parchment paper. The coating process involved two steps.^33^ First, a cationic starch solution with or without OmEO was prepared. Last, the solution was applied onto the surface of the paper. Cationic starch was dissolved in distilled water (5.0%, w/w) at 90–95 °C under a constant stirring for 10 min. Its viscosity was 37 cP determined at 75 °C at 100 rpm using a Brookfield DV-II + Viscometer, with spindle no. 2. The temperature was then lowered to 70 °C, during which 10% OmEO (on dry matter basis of cationic starch, w/w) was added at constant stirring of 2000 rpm for 10 min. The viscosity of OmEO and starch mixture was 31 cP at 75 °C. The half of cationic starch solution containing no OmEO was kept for the production of control paper. The final solutions with 60 °C temperature were applied onto the surface of paper using a paper coating applicator (model K202, RK Print Coat Instruments, company reg. no.: 775106, UK) with 3 cm/s speed. Two different coated papers were produced; the control paper was coated with cationic starch only and the test paper was coated with cationic starch + OmEO. All the paper was kept at 23 °C under 50% humidity until packaging.^33^

### Infrared Spectroscopic (ATR-FTIR) Measurements of Papers

In the study, 10 homogeneous samples obtained from the control and test papers were prepared for spectrum measurement using an ATR-FTIR device (Bruker OPUS). A measurement was performed by placing a new sample on a cleaned ATR-FTIR sample plate at each time. A spectrum was first collected and then immediately corrected for a background spectrum by co-adding 64 scans at every 2 cm^–1^ frequencies over a wavelength of 4000 to 400 cm^–1^. All the collected spectra (totally 20 spectra, 10 by 2 paper samples) were subjected to a linear baseline correction and peak absorbance normalization by rescaling according to the highest peak value of the region and smoothened with 9-points without distorting the peak location and height using a Spectragryph software for optical spectroscopy version 1.2.14, kindly provided by Dr. Friedrich Menges, Germany. The normalized spectra data was analyzed for partial least square regression analysis (PLSR) over a range of 1800 to 800 cm^–1^ of the finger print region. The number of components was set at a maximum of 10 with no cross-validation. To differentiate OmEO-coated paper from the control paper, a PLSR score plot with three components employing FTIR spectrum data was generated using MINITAB 16 (Minitab, Inc., USA).

### Scanning Electron Microscopy (SEM)

Morphological characterization was performed using a JSM-6510 microscope (JEOL Ltd., Japan) running at 5 kV and a working distance of 2–3 mm.

### Meat Packaging

Meat was obtained from a 22 month-old Simmental bull finished in a feedlot for 6 months in Karaman, Turkey. Immediately after slaughtering, its carcass was chilled at +4 °C according to national food safety regulation.^34^ The meat was a longissimus dorsi muscle from the seventh to the last lumbar vertebra. The meat was then minced using a sterilized mincing machine with a 6 mm sieve diameter. The minced meat was gently mixed in aseptic condition and kept in ice until the preparation of test samples. Sterilized glass Petri dishes (121 °C for 15 min) of 100 mm in diameter and 20 mm in height were randomly placed with seven to nine pieces of homogenized minced meat. Each piece of meat on Petri dishes had a diameter of 15 mm and a height of 15 mm, obtained using an apparatus specially designed and produced by a 3D printer. A total of 12 Petri dishes were randomly covered by the coated papers and then immediately tightened up by placing the upper glass lid over the coated paper to simulate a general practice of minced meat packaging in the Turkish market. Thus, a 0.5 mm headspace between the top of meat pieces and the coating paper was ensured on all dishes. The meat samples were then stored at +4 °C in an aseptically cleaned laboratory refrigerator for 0, 6, and 12 days, similar to those in market conditions. All physical (color parameters), chemical, and microbiological tests were carried out on six replicates per sample at each storage period.

### Texture, Color Parameters, Dry Matter (DM), Water Activity (*A*_w_), and Sensory Analysis

In this study, beef meat was subjected to a mincing process that caused muscle fiber damage. Therefore, texture analysis was only conducted using first compression to determine the hardness (N/cm^2^) only, which is the “maximum force required to compress the sample”. The device used to measure the hardness was a TA.XT.Plus Texture Analyser running under the conditions of a pretest speed of 1.0 mm s^–1^, a test speed of 2 mm s^–1^, a post-test speed of 10.0 mm s ^–1^, a head speed of 2.0 mm s^–1^, a distance of 5.0 mm, and a force of 5 g (Stable Microsystem, Surrey, United Kingdom). Minced beef subjected to compression force had a size of 15 mm in diameter and 15 mm in height. In each of experimental treatment at every storage period, six independent measurements were taken.

A Color Flex s/n CX2733 HunterLab date 5-10 model (Hunter Associates Laboratory, Reston, VA, USA) device with a black-white calibration tile was used to measure the color parameters L* (brightness), a* (green to red), and b* (blue to yellow).^34^ The hue angle (tonality) [180/π × arctan × (b*/a*)] and Chroma (saturation index) [(a*^2^ + b*^2^)^1/2^] were calculated. Dry matter (DM) of samples was determined by using an automoisture analyzer (OHAUS MB45, Switzerland).^35^ The water activity (*A*_w_) was determined by using an auto-analyzer device (Novasin, LabMASTER).^35^

Sensory evaluation was carried out by a trained group of Faculty of Engineering at Karamanoglu Mehmetbey University, Turkey, comprised of 10 persons (5 females and 5 males), who were pretrained on various commercially available fresh minced beef products to test only their color and odor. On each storage period, six Petri dishes of control and OmEO groups were randomly introduced to each person at different time intervals. A 1.0 to 5.0 grading system was used to evaluate the color (5, reddish brown; 4, bright red; 3, pinkish red; 2, pink; 1, pale pink) and odor (1, very unpleasant; 2, unpleasant; 3, acceptable; 4, pleasant; 5, very pleasant).

### pH and HCl Titration Value, Peroxide Value (PV), Thiobarbituric Acid Reactive Substances (TBA), and DPPH Scavenging Activity

pH was measured in 10 g of homogenized meat in 100 mL of distilled water by using a pH meter. To determine pH-related meat spoilage by HCl titration, the homogenized meat in distilled water (10:100, v:v) was filtered through a filter paper and the collected aliquot was homogeneously mixed at 150 rpm for 2 min. The initial pH of homogenate was recorded. Two milliliters of aliquot was titrated with 0.02 N HCl to lower the initial pH to a pH value of 5.0 using an automated titration device. The amount of HCl required to obtain a pH value of 5.0 was then calculated as mL HCl titrate per gram of sample. The results were then evaluated as follows: the higher the HCl titrate value, the higher the degree of pH-dependent meat spoilage.

The peroxide value (PV) of the meat sample was analyzed according to the method of Nizamlioglu.^35^ Briefly, 5 g of sample was immersed for 2 min in 30 mL of chloroform in the presence of anhydrous sodium sulfate. The mixture was filtered through Whatman filter paper (no. 1), and 25 mL of aliquot chloroform extract was vigorously mixed with 30 mL of glacial acetic acid and 2 mL of saturated potassium iodide solution. After 2 min, 100 mL of distilled water and 2 mL of fresh 1% starch solution were added to the content. The final solution was titrated immediately with 0.1 N sodium thiosulfate to obtain a colorless solution. The PV was calculated as mEq/kg sample (0.1 × mL 0.1 N sodium thiosulfate/sample weight ×100).

The extent of lipid oxidation in different samples was assessed by the method reported by Koniecko^36^ through the determination of thiobarbituric acid reactive substances (TBA) of malondialdehyde (MDA). Briefly, 10 g of sample was homogenized in 50 mL of distilled water for 2 min. An additional 47.5 mL of distilled water and 2.5 mL of 4 N HCl to lower the pH to 1.5 were added. A total of 100 mL solution containing the sample was distilled until the collection of 50 mL of distillate. Five milliliters of final distillate was mixed with 5 mL of thiobarbituric acid (TBA) reagent (5 mM) in a test tube and cooled in running tap water after boiling in a water bath at 70 °C for 35 min. The absorbance was measured at a fixed wavelength of 538 nm using a spectrophotometer. The TBA value was calculated as milligrams of malonaldehyde per kilograms of the sample by multiplying the absorbance value with a factor (7.8).

Free radical scavenging activity was evaluated by using stable radical 2,2-diphenyl-1-picrylhydrazyl (DPPH) according to method used by Martins et al.^37^ Five grams of sample mixed with 25 mL of methanol was homogenized at 20.000 rpm for 30 second using an Ultra-Turrax T25 device at room temperature. The sample was centrifuged at 7200 rpm for 10 min at +4 °C. The sample was then filtered through a Whatman no. 1 paper. The sample was mixed with DPPH solution, its absorbance was measured at 517 nm by using a PerkinElmer spectrophotometer, and DPPH free radical scavenging activity was calculated as follows: DPPH radical scavenging activity (%) = (1 – absorbance of sample/absorbance of control, DPPH) × 100.

### Aerobic Psychrophilic Bacteria Count (APC)

The aerobic psychrophilic bacteria count (APC) of samples from different treatment groups was determined at different storage intervals by using the pour plate method as described by ICMSF.^38^ Ten grams of meat sample was homogenized in 90 mL of sterile peptone water (0.1%). A serial dilution up to 10^–9^ was prepared using sterile peptone water and then duplicate-plated with plate count agar. All the samples were then incubated at +4 °C for 10 days. Microbial colonies from the plates were counted and expressed as the logarithmic colony forming unit (Log 10 CFU/g).

### Statistical Analysis

Experimental design contained two treatments (control and OmEO) by three storage periods (0, 6, and 12 days) by six batches by six measurements per physical, chemical, and microbiological parameters. The data was subjected to an analysis of variance using General Linear Model under MINITAB 16 (Minitab, Inc., USA). Significant differences between the means were separated using Tukey’s multiple comparison test. The results were expressed as mean ± Stdev (standard deviation), and the significance level was set at *P* < 0.05. A principle component analysis (PCA) was carried out on all the physical, chemical, and microbiological data to differentiate between the meat products treated with or without OmEO. A PCA generated a biplot based on eigenvalues and a dendogram based on correlation coefficients using a cluster analysis according to a complete-linkage method to demonstrate the degree of similarities between the analyzed parameters.

## Results and Discussion

### Chemical Composition

GC/MS spectrometric analysis showed that OmEO contained 0.82% α-pinene + α-thujene, 0.10% camphene, 0.07% β-pinene-2, 0.62% β-myrcene, 0.12% α-phellanderene, 0.57% α-terpinene, 0.10% limonene-*d*, 0.12% β-phellanderene, 1.92% γ-terpinene, 2.21% *p*-cymene, 0.08% α-terpinolene 17.50% linalool, 0.69% terpinene-4-ol, 1.01% β-caryophyllene, 0.71% α-terpineol, 0.56% borneol, 0.95% thymol, 69.26% carvacrol, 0.09% β-bisabolene, and 2.73% undefined. OmEO used in our study has high carvacrol content and low thymol content. These results were almost similar to the results of Rodriguez et al.^3^ reported for OmEO obtained from the same species grown on Mediterranean region (Mersin) of Turkey.

### SEM and FTIR Spectroscopy Analysis

**Figure 1:**
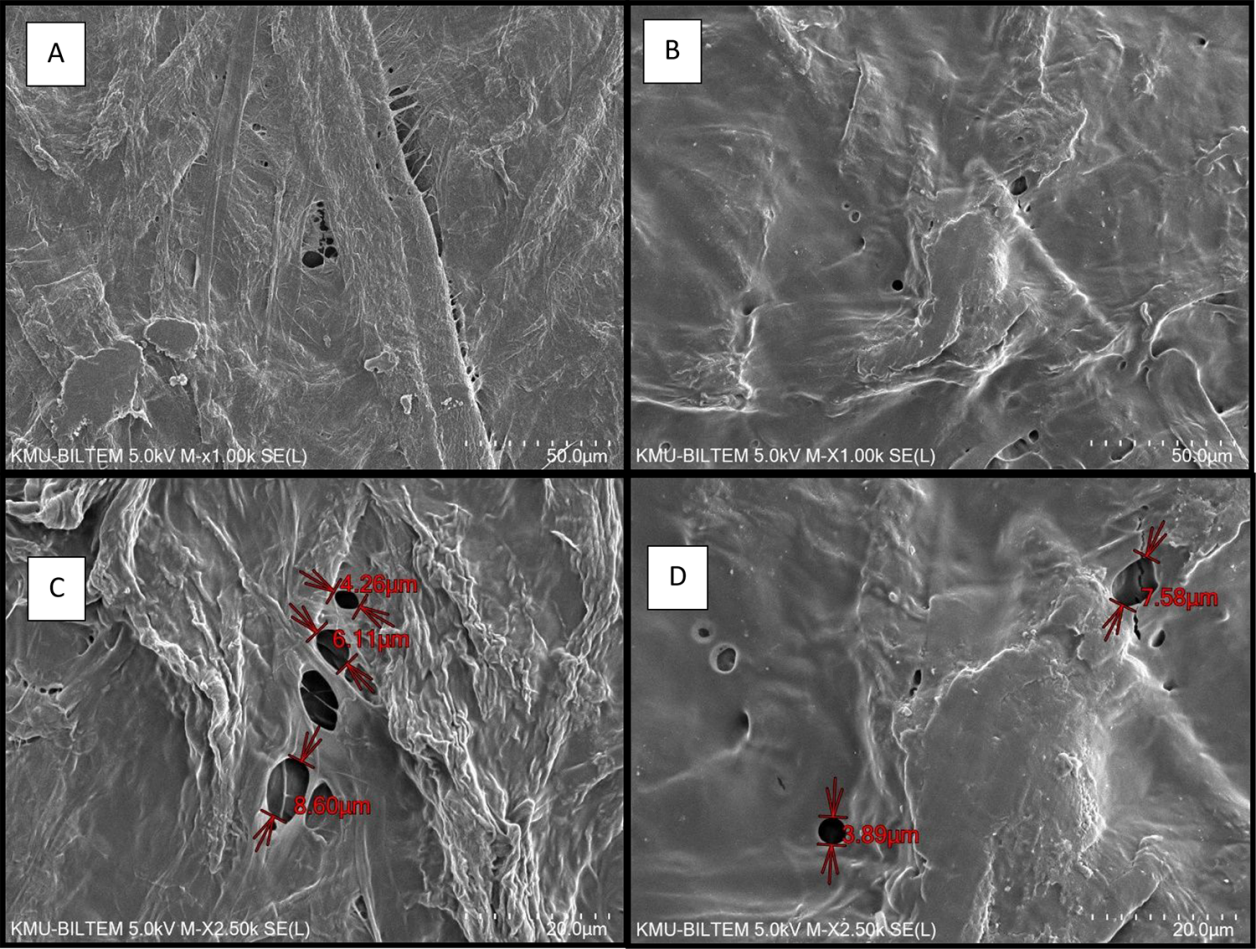
Scanning electron microscopy (SEM) micrographs of the surface and cross sections of (A, C) the control paper and (B, D) OmEO-coated paper.

Figure 1 shows that the paper coated with OmEO (Figure 1C) had very smooth, compact, and continuous surface and presented a homogeneous and cohesive internal structure in comparison to the control paper (Figure 1A). In addition, OmEO was homogeneously distributed over the paper surface, providing fewer irregularities, a few holes with low porosity, and a bubble-like structure, indicating the OmEO presence (Figure 1C,D). The control paper had a rough surface, an intense irregularity, and an increased number of holes with high porosity (Figure 1B). These results showed that OmEO immobilized on the surface. OmEO contained an appreciable amount of volatile and nonvolatile compounds with hydrophobic characteristic, namely, phenols, terpenes, and aldehydes. In our study, cationic starch was used to emulsify OmEO to act as a surfactant. SEM pictures showed that the cationic starch acted a good carrier of OmEO and evenly distributed OmEO on the surface of the paper to reveal its antimicrobial and antioxidant effect during storage of minced beef in our study. Similar results were reported with a cationic surfactant (lauric arginate) carrying TEO^39^ and with active packaging pectin films with OmEO.^14^

**Figure 2:**
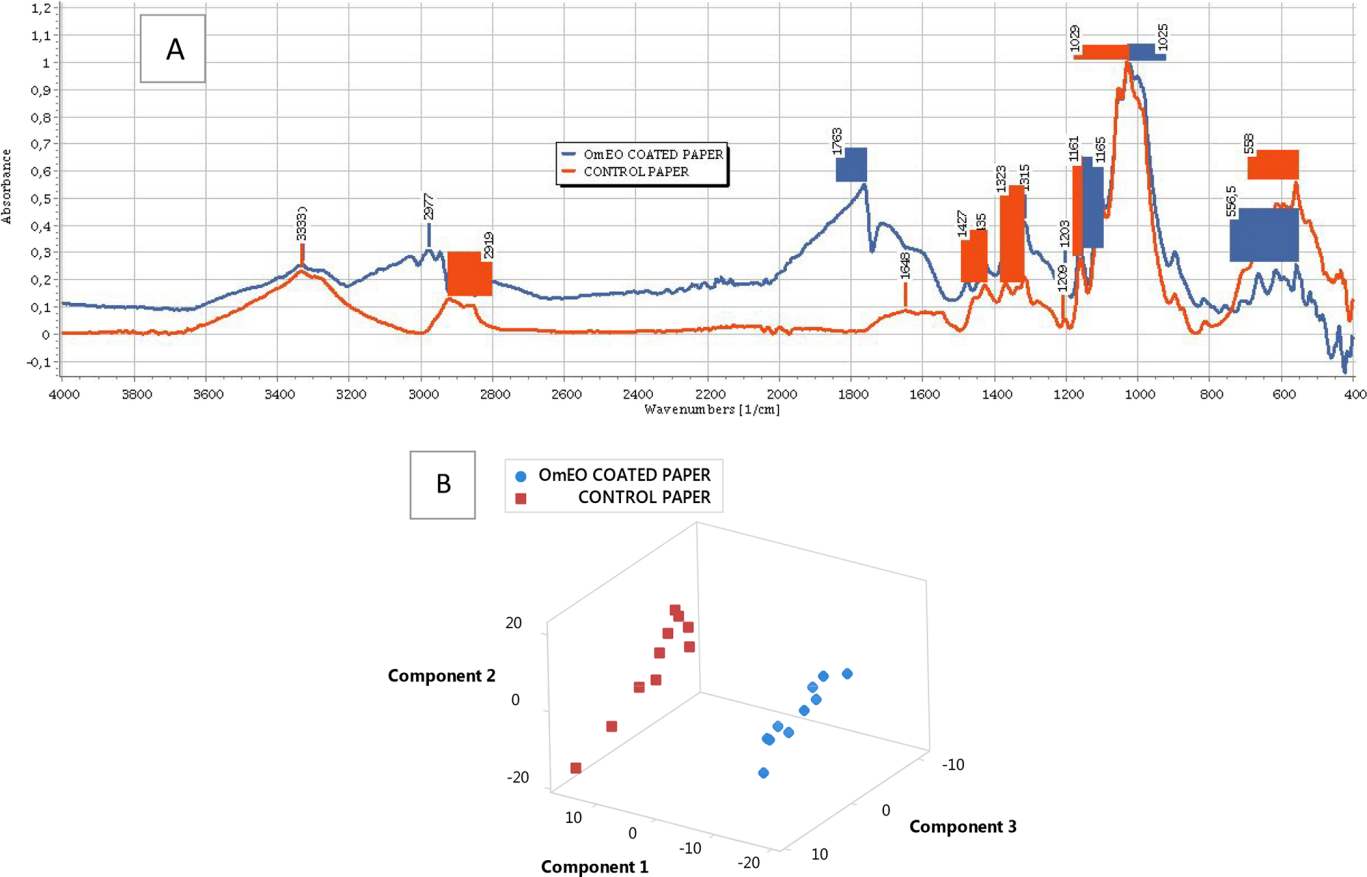
(A) ATR-FTIR spectra (0 to 1 scale normalization applied after baseline correction) of control paper and OmEO-coated paper at a range of 4000 to 400 cm^–1^ wavelength and (B) score plot showing a discrimination between control paper and OmEO-coated paper. Ten independent spectrum measurements were recorded per each paper.

Significant intermolecular and structural chemical changes were observed in the cationic starch-OmEO-coated paper compared to the control papers coated only with cationic starch when ATR-FTIR spectra examined in detail (Figure 2A). The bands of 3400 and 3200 cm^–1^ are related to −OH stretching vibration, the band of 3000–2800 cm^–1^ is for −CH stretching, the ester bonding (C=O) in the range of 1800–1700 cm^–1^ is for carbonyl group stretching vibration, the amide I region at 1645 cm^–1^ is for −OH or −NH bending, the band of 1500–1300 cm^–1^ is for variable −CH bending vibrations, the band of 1380–1080 cm^–1^ is for −CN stretching vibration, and the band of 1200–900 cm^–1^ is for −CO and −COC ester and glycoside bonds referring to polysaccharides. In comparison to the control paper coated only with cationic starch, OmEO coating caused remarkable stretching and bending vibrations at the region of 3000 to 2800 cm^–1^, where there were new stretching peaks of −CH, −CH_2_, and CH_3_ groups, which are largely present in OmEO. The presence of similar peaks was earlier reported with similar plant oils.^10,15,40^ Moreover, a new and sharp peak at 1763 cm^–1^ is an indication of intense carbonyl group formation due to SM/AG present in OmEO. The stretching and bending vibrations of carbonyl, vinyl, methyl, and methylene groups are demonstrated to act as identifiers of EO in the ATR-FTIR spectra, and a sharp peak at around 1745 cm^–1^ indicates the presence of a wide range of carbonyl, vinyl, methyl, and methylene compounds in EO products.^41^ There were stretching and bending vibrations at the region of 1700 to 900 cm^–1^, which are stronger in OmEO-coated paper than those in the control paper. In terms of the absorbance under the area of peaks shown in Figure 2A, the absorbance at 3330 cm^–1^ is lower in OmEO paper than in the control paper. A similar change on this band was previously reported^42^ when TEO^39^ is introduced into hydroxypropyl methylcellulose films. On the other hand, the absorbance under the peaks from 1800 to 1200 cm^–1^ band is higher in OmEO than in the control paper. These changes were possibly due to phenolic and aromatic compounds of OmEO entrapped within the intermolecular structure of the paper since the phenolic compound of EO has been reported to have its own characteristic peaks at the band of 1800 to 1200 cm^–1^.^43^ Similar molecular and structural changes were recently reported on starch films containing TEO^29^ and on the nanofiber produced from eugenol EO immersed in cationic starch.^30^ In our study, a score plot of all the absorbance values from 1800 to 800 cm^–1^ wavelength region (Figure 2B) clearly indicated that the paper coated with OmEO has a clear and distinctive chemical conformation and structure, which markedly differed from the control paper.

### Changes in Color Parameters during Cold Storage

The lightness (L*) value of minced beef (Table 1) was significantly affected by coating treatments (*P* = 0.037) and by the storage period (*P* = 0.001) but not by their interaction (*P* = 0.314). The lightness (42.20 ± 3.67) of minced meat packaged with OmEO was higher than that (40.70 ± 4.86) of minced meat packed without OmEO. The L* value gradually decreased from 42.22 ± 0.71 at the 0th day to 40.95 ± 2.69 at the 6th day of storage and to 37.16 ± 2.52 at the 12th day of storage. On the other hand, a* (redness), b* (yellowness), Hue angle (tonality), and Chroma values (saturation) were insignificantly affected by the paper coating treatments and the interaction of paper coating treatments by storage period, while the effect of storage period was significant (*P* = 0.001) on all parameters. Increasing the days of storage caused decreased redness and yellowness. The Hue angle represents the full spectrum of color and exists from 0° to 360°.^44^ This means that minced beef has a Hue angle of around 47.5°, indicating a golden yellow color (khaki color) at the 0th day, which gradually reduced to a value of 28° with an orange-red color (vermilion). The higher the Hue angle, the lesser the red color of the product. The same case was applied to the Chroma value, which was lowered from a value of 30 at the 0th day of storage to a value of 17 at the 12th day of storage, meaning that there is a washed and less pure color in pastels of meat. The redness of the meat product tends to reduce by an increase in storage time, during which a conversion of red oxymyoglobin into metmyoglobin occurs, and this may be responsible for the development of a brown/reddish color.^40^ The changes in color parameters by the days of storage in all treatment groups in our study were found to be similar to the results reported previously, with red meat coating with an edible film containing EO^6,40^ but a degree of reduction in color parameters, especially in the L* value, which is comparatively low with all EO-treated groups. However, some other studies demonstrated that mixing plant EO with the meat products with or without inoculation of pathogen bacteria or coating meat with edible films containing EO kept all the color parameters almost unaffected or prevented the color losses during storage at 4 °C.^9,22,23,45,46^ In these studies, the meat coated with an edible film containing no EO even provided a protection of the meat color. Thus, edible coating films may exhibit a water-barrier property and retain water in the product.^9^ In fact, mixing or coating the meat with EO fully covered the entire meat surface, and the antioxidant effects of EO become highly effective for the prevention of color losses, although the odor and other sensory parameters may have been worsened. On the other hand, the meat with active packaging as in our study does not cause any negative impacts on the sensory quality of meat since the meat was only subjected to the protection of the active substance of EO available on the headspace, and the magnitude of the effectiveness of paper coating or active packaging on the meat color parameters may comparatively be considered low.^28^

**Table 1:** Changes in Physical and Sensorial Parameters (Mean ± Stdev, Mean with Standard Deviation) of Minced Meat Influenced by the Paper Coating Treatment and the Days of Storage at 2 °C^b^

	physical parameters					sensory parameters^a^	
	L* (lightness)	a* (redness)	b* (yellowness)	Hue angle (tonality)	Chroma (saturation)	color	odor
treatment							
control	40.70 ± 4.86^b^	13.84 ± 6.68^a^	17.56 ± 2.68^a^	36.07 ± 8.76^a^	22.63 ± 6.25^a^	4.35 ± 0.93^a^	2.53 ± 0.98^b^
OmEO	42.20 ± 3.67^a^	13.94 ± 6.65^a^	17.78 ± 2.57^a^	36.02 ± 8.50^a^	22.85 ± 6.10z^a^	3.42 ± 0.67^b^	3.58 ± 0.60^a^
days of storage							
0 day	46.22 ± 0.71^a^	22.78 ± 1.10^a^	20.87 ± 0.68^a^	47.51 ± 0.56^a^	30.90 ± 1.26^a^	2.97 ± 0.18^b^	4.00 ± 0.00^a^
6 days	40.95 ± 2.69^b^	10.57 ± 0.97^b^	17.30 ± 0.53^b^	31.39 ± 2.32^b^	20.20 ± 0.73^b^	4.02 ± 0.99^a^	3.00 ± 1.06^b^
12 days	37.16 ± 2.52^c^	8.31 ± 0.89^c^	14.83 ± 0.72^c^	29.23 ± 2.60^c^	17.02 ± 0.85^c^	4.16 ± 0.80^a^	2.66 ± 0.75^c^
interaction							
control, 0 day	46.22 ± 0.75^a^	22.81 ± 1.12^a^	20.87 ± 0.72^a^	47.54 ± 0.53^a^	30.92 ± 1.30^a^	3.00 ± 0.05^c^	4.00 ± 0.00^a^
OmEO, 0 day	46.20 ± 0.55^a^	22.76 ± 1.02^a^	20.87 ± 0.67^a^	47.48 ± 0.65^a^	30.88 ± 1.33^a^	2.99 ± 0.25^c^	4.00 ± 0.00^a^
control, 6 days	39.91 ± 3.06^bc^	10.36 ± 1.33^b^	17.08 ± 0.31^b^	31.17 ± 3.12^b^	20.00 ± 0.80^b^	4.87 ± 0.46^a^	2.12 ± 0.79^c^
OmEO, 6 days	41.98 ± 2.01^b^	10.77 ± 0.45^b^	17.53 ± 0.63^b^	31.61 ± 1.43^b^	20.58 ± 0.56^b^	3.17 ± 0.55^c^	3.88 ± 0.33^a^
control, 12 days	35.92 ± 2.47^d^	8.34 ± 1.10^c^	14.72 ± 0.86^c^	29.50 ± 3.47^b^	16.95 ± 0.94^c^	4.33 ± 0.96^b^	2.33 ± 0.75^c^
OmEO, 12 days	38.36 ± 2.12^cd^	8.28 ± 0.73^c^	14.94 ± 0.61^c^	28.97 ± 1.60^b^	17.09 ± 0.83^c^	4.00 ± 0.58^b^	3.00 ± 0.58^b^
level of significance (*P*)							
treatments	0.037	0.771	0.331	0.944	0.507	0.001	0.001
days	0.001	0.001	0.001	0.001	0.001	0.001	0.001
interaction	0.314	0.815	0.719	0.856	0.747	0.001	0.001

a Following grading systems used during sensory evaluation: color (5, reddish brown; 4, bright red; 3, pinkish red; 2, pink; 1, pale pink) and odor (1, very unpleasant; 2, unpleasant; 3, acceptable; 4, pleasant; 5, very pleasant).

b Mean values given with different superscripts (a to d) within the same column group were statistically (*P* < 0.05) different.

### Sensory and Texture Evaluation

Minced beef subjected to experimental treatments was evaluated for color and odor by a sensory panel (Table 1). Irrespective of days of storage, meat color remained in pinkish red color with the OmEO paper group, while the color of the control paper group was evaluated as bright red. This difference was found significant (*P* = 0.001). Similarly, the odor of the meat in the OmEO group was evaluated as acceptably pleasant, while the meat of the control group was evaluated as unpleasant, and the difference between both groups was also significant (*P* = 0.001). The effects of storage period and the interaction of active coating by storage were significant on both color and odor parameters. Increasing the period of storage gradually changed the color of meat from pinkish red to red or reddish brown and the odor from pleasant to unpleasant. But these changes during the storage period varied differently between the control and OmEO-coated paper groups. The meat of the control group significantly lost its color and odor from a pinkish red color with pleasant odor at the 0th day to a reddish-brown color with unpleasant odor at the 6th day of storage and bright red color with unpleasant odor at the 12th day of storage, while the OmEO-coated meat remained its color either in pinkish red with pleasant odor at the 6th day of storage and in bright red with acceptable odor at the 12th day of storage. These sensed results confirmed the changes in measured color parameters in our study. OmEO has a strong odor and aroma, which may modify meat organoleptic properties. For instance, in minced beef products, a mixing rate of EO of up to 0.6% was found suitably sufficient with no negative alteration in sensory parameters.^47^ It was previously reported that the TEO and oregano EO used in meat contact and packaging materials reduced the color losses during cold storage and the products have a general acceptability by the consumer.^7,17,19,22^

In our study, OmEO successfully provides protection against the sensed color and odor losses, particularly until the 6th day of cold storage, and there were not any signs of undesirability in color and odor of minced beef with OmEO despite the fact that the control meat that changed in sensed odor and color was unfit for consumption at the 6th day of storage.

Active packaging of minced beef with the paper coated with OmEO in cationic starch significantly (*P* = 0.040) affected the hardness (Table 2). The effect of storage period was significant on the meat hardness (*P* = 0.003) and the interaction effect of paper coating by storage period was also significant (*P* = 0.031). The hardness of meat was increased from 0.59 N/s on the 0th day to 0.97 N/s on the 6th day of storage in the control group, while the magnitude of increased meat hardness was found smaller in the OmEO group at the 6th day of storage (an increase from 0.58 to 0.70 N/s). On the 12th day of meat storage, the differences between OmEO and control groups were not significant, and the meat hardness of both groups reduced up to 0.60 to 0.68 N/s, which was not significantly different from the values on the 0th day. These results indicated that OmEO emulsified in cationic starch maintained the hardness of meat up to the 6th day of storage. Similarly, it has been reported that lemon/thyme EO-enriched chitosan coating maintained the 0th day’s hardness and color and retarded lipid/protein oxidation and microbial growth in grass carp filled during cold storage.^48^ It was reported that the meat wrapped up with nanofiber of eugenol EO with cationic starch kept its hardness up to the 5th day of storage at 4 °C, while adhesiveness, cohesion, springiness, and chewiness parameters remained unaffected.^30^

**Table 2:** Changes in Hardness, pH, HCl Titrate, DM, *A*_w_, and APC (Mean ± Stdev, Mean with Standard Deviation) Affected by OmEO Paper Coating^a^

	hardness (N/cm^2^) (mean ± Stdev)	pH (mean ± Stdev)	HCl titrate value (mL/g) (mean ± Stdev)	DM (%) (mean ± Stdev)	*A*_w_ (mean ± Stdev)	APC (Log 10 CFU/g) (mean ± Stdev)
treatment**s**						
control	0.75 ± 0.22^a^	6.78 ± 0.90^a^	0.96 ± 0.65^a^	43.50 ± 11.0^a^	0.96 ± 0.01^a^	5.68 ± 0.51^a^
OmEO	0.63 ± 0.16^b^	6.56 ± 0.84^b^	0.70 ± 0.55^b^	40.85 ± 8.90^a^	0.96 ± 0.01^a^	3.93 ± 0.38^b^
days of storage						
0 day	0.59 ± 0.20^b^	5.64 ± 0.01^c^	0.10 ± 0.01^c^	34.39 ± 3.00^b^	0.96 ± 0.02^a^	2.43 ± 0.02^ab^
6 days	0.84 ± 0.16^a^	6.68 ± 0.26^b^	0.86 ± 0.30^b^	38.63 ± 5.20^b^	0.95 ± 0.01^a^	4.63 ± 0.01^b^
12 days	0.65 ± 0.17^b^	7.68 ± 0.26^a^	1.52 ± 0.11^a^	53.50 ± 7.85^a^	0.97 ± 0.03^a^	7.37 ± 0.03^a^
interaction						
control, 0 day	0.59 ± 0.21^c^	5.64 ± 0.01^d^	0.10 ± 0.01^e^	34.39 ± 3.15^b^	0.96 ± 0.02^a^	2.47 ± 0.11^d^
OmEO, 0 days	0.58 ± 0.23^c^	5.63 ± 0.01^d^	0.10 ± 0.01^e^	34.41 ± 2.85^b^	0.96 ± 0.02^a^	2.40 ± 0.32^d^
control, 6 days	0.97 ± 0.07^a^	6.94 ± 0.03^b^	1.16 ± 0.04^c^	40.38 ± 6.15^b^	0.95 ± 0.01^a^	5.85 ± 0.31^b^
OmEO, 6 days	0.70 ± 0.09^b^	6.43 ± 0.01^c^	0.56 ± 0.01^d^	36.87 ± 3.78^b^	0.96 ± 0.01^a^	3.41 ± 0.56^c^
control, 12 days	0.68 ± 0.16^bc^	7.76 ± 0.28^a^	1.62 ± 0.03^a^	55.71 ± 8.84^a^	0.97 ± 0.01^a^	8.73 ± 0.12^a^
OmEO, 12 days	0.60 ± 0.09^c^	7.61 ± 0.25^a^	1.41 ± 0.02^b^	51.29 ± 6.77^a^	0.96 ± 0.01^a^	6.01 ± 0.33^b^
level of significance (*P*)						
treatments	0.040	0.001	0.001	0.175	0.560	0.001
days	0.003	0.001	0.001	0.002	0.200	0.001
interaction	0.031	0.001	0.001	0.610	0.067	0.001

a Mean values given with different superscripts (a to e) within the same column group were statistically (*P* < 0.05) different.

### Changes in pH, DM, *A*_w_, HCl Titrate Value, and APC

During cold storage, meat and meat products inevitably undergo some chemical and microbiological changes, causing meat spoilage and deterioration. In our study, OmEO-coated paper remarkably delayed microbial and chemical deterioration up to 12 days of cold storage. Overall, OmEO-coated paper caused a significant (*P* = 0.001) reduction in pH by 3.2% from 6.78 ± 0.90 in control meat to 6.56 ± 0.84 in OmEO meat, HCl titrate value by 27%, and microbial growth by 1.75 Log 10 CFU/g and led to an insignificant (*P* = 0.175) but numerical decrease in DM loss by 6.8%. There were however sporadic changes in *A*_w_ values (0.96 ± 0.02, 0.95 ± 0.01, and 0.97 ± 0.03 at 0th, 6th, and 12th days of storage, respectively) of minced beef, which were not significantly affected by the treatments. During cold storage, the pH of minced meat significantly (*P* = 0.001) increased from 5.64 ± 0.01 at the 0th day to 7.68 ± 0.26 at the 12th day of storage, HCl titrate value from 0.10 ± 0.01 to 1.52 ± 0.11, dry matter from 34.39 ± 3.00 to 53.5 ± 7.85%, and APC from 2.43 ± 0.02 to 7.37 ± 0.03 Log 10 CFU/g. Except in the case of DM, these parameters at each storage period significantly (*P* = 0.001) differed between OmEO- and non-OmEO-coated papers, although DM values in each storage period were numerically lower in the OmEO group paper than those in the control paper group. None of the parameters significantly differed between the control and OmEO papers at the 0th day of storage. On the other hand, the difference in meat pH between the control and OmEO papers was significant only at the 6th day of storage (6.43 ± 0.01 in OmEO versus 6.94 ± 0.03 in control). During the 12th day of storage, the meat of both groups reached a similar pH value of 7.68 ± 0.26. However, HCl titrate values in the OmEO paper group at 6th and 12th days of storage were 0.56 ± 0.01 and 1.41 ± 0.02, which were significantly (*P* = 0.01) lower than the values of 1.16 ± 0.04 and 1.62 ± 0.03 in the control group, respectively. Similar changes in microbial growth (APC value) were observed during storage. In comparison to non-OmEO-coated paper, OmEO paper markedly reduced microbial growth at 6th and 12th days of storage, and the reduction rates were about 2.44 and 2.72 Log 10 CFU/g of APC, respectively (Table 2).

### DPPH Scavenging Activity, PV, and TBA Values of Minced Meat

**Figure 3:**
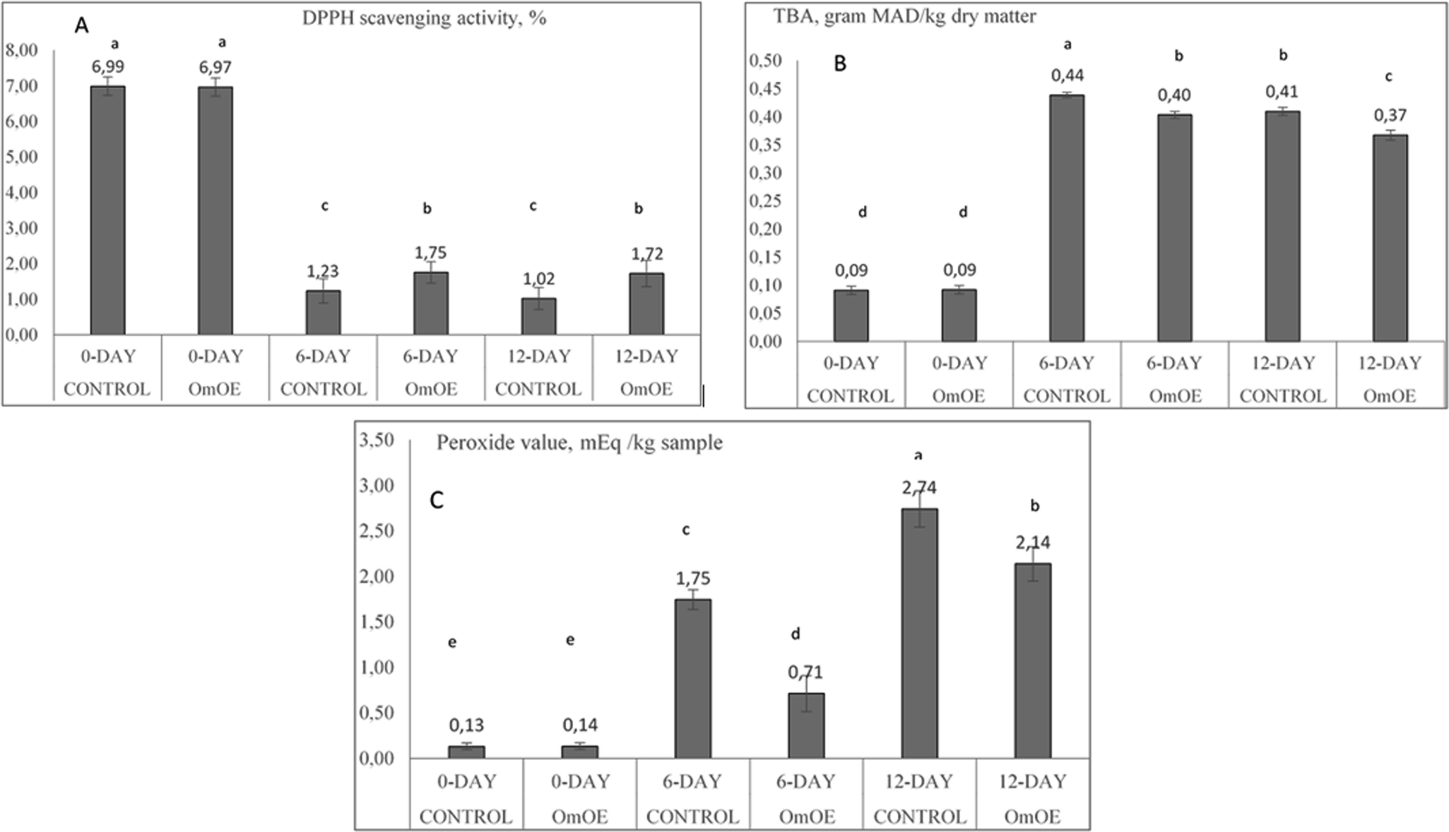
DPPH scavenging activity (%), degree of lipid peroxidation (TBA g MAD per kg sample), and peroxide value (mEq/kg sample) of the minced meat packed with active paper coated with or without OmEO. Significant (*P* < 0.05) differences between the means with Stdev are shown by different letters.

DPPH scavenging activity (%), TBA (g MDA per kg), and PV (mEq/kg) were significantly (*P* < 0.05) affected by the paper coating, days of storage, and their interaction (Figure 3A). DPPH scavenging activity markedly reduced from 6.98 ± 0.25 at the 0th day to 1.50 ± 0.32 at the 6th day and to 1.37 ± 0.34 at the 12th day of storage. DPPH scavenging activity values of 1.75 ± 0.30 and 1.72 ± 0.37% at 6th and 12th days of storage were significantly higher in the OmEO group than the values of 1.23 ± 0.34 and 1.02 ± 0.31% of the control group, respectively. However, there was an overall 40% higher DPPH scavenging activity of meat in the OmEO paper group at the 12th day of storage.

TBA values of meat at the 0th day of storage in control and OmEO groups were around 0.092 ± 0.01 g MDA/kg, and the difference in TBA of both groups was insignificant (Figure 3B). However, these values significantly (*P* < 0.05) increased to 0.42 ± 0.0055 and 0.39 ± 0.008 g MDA/kg at 6th and 12th days of storage, respectively. TBA values of 0.40 ± 0.006 g MDA/kg at the 6th day and of 0.37 ± 0.009 g MDA/kg at the 12th day of storage in the OmEO group were significantly (*P* < 0.05) lower than the values of 0.44 ± 0.005 and 0.41 ± 0.007 g MDA/kg in the control group, respectively. Thus, the TBA value of meat in OmEO-coated paper was 10% lower than that of meat in non-OmEO-coated paper at the 12th day of storage.

PV (mEq/kg) is a direct indication of rancidity of unsaturated fats and oils, indicating how much peroxide or toxic substances are formed. In sensory analysis, a strange flavor or odor is being noticed in the rancid meat and meat products. In our experiment, minced beef has a low level of PV of 0.134 ± 0.036 mEq/kg sample at the 0th day of storage, which was significantly (*P* < 0.05) raised up to levels of 1.747 ± 0.111 and 2.743 ± 0.200 mEq/kg sample at 6th and 12th days of cold storage of the control meat (Figure 3C). On the other hand, the corresponding PV values of OmEO-coated paper remained lower at 0.713 ± 0.197 mEq/kg sample at the 6th day and 2.140 ± 0.190 mEq/kg sample at the 12th day of storage. Therefore, it can be said that the magnitude of increased PV was significantly (*P* < 0.05) low with OmEO treatment in our experiment since there was a 22% decrease in the PV of meat sample at the 12th day of storage by OmEO treatment.

Meat coated with edible films containing TEO was kept under modified atmosphere packaging (MAP) for 1, 3, 7, and 10 days at 2 and 4 °C. In comparison to the control meat samples, TEO caused lower values of lipid oxidation, DM losses, and pH, better color (lower lightness but higher redness and yellowness), and increased antioxidant capacity.^22^ Similar results with edible films containing TEO were earlier reported by Vital et al.^23^ and Guerrero et al.^9^ These results were similar to the results obtained from our study. In these studies, a strong odor of TEO largely penetrated into the meat throughout the storage period due to the direct contact of TEO with meat, and the consumer acceptability of TEO-treated meat is presumably limited. In our study, OmEO did not directly mix with meat and rather was entrapped in the headspace in the package. The color and odor of minced beef with OmEO during cold storage in our study were evaluated as acceptably pleasant.

Chang et al.^39^ determined the strong antimicrobial activity of a nanoemulsion of TEO with a cationic surfactant. A nanofiber produced from eugenol EO with cationic starch reduced the growth of *Bacillus cereus* by 2.0 Log 10 CFU/g of beef meat stored at 4 and 25 °C for 5 days without altering the meat color and texture.^30^ In another study,^29^ TEO-starch films were shown to have an inhibitory action against *Botryodiplodia theobromae* Pat. and *Colletotrichum gloeosporioides* Penz. Similarly, microemulsifying TEO films in minced meat products have high antimicrobial efficacy against coliforms, *Staphylococcus aureus*, yeast, mold, and lactic acid bacteria.^10^ Coating films with TEO together with chitosan or mixing of the meat product with thyme microcapsules was shown to have great antifungal and antibacterial activity in fermented and smoked sausages.^8,11,20^ Mixing meat with EO and microencapsulated EO is also reported to have strong antimicrobial activity in the meat.^7,11^ All these findings supported our results. OmEO with cationic starch was very effective against the microbial growth of APC, providing an overall of 1.75 Log 10 CFU/g reduction in the minced meat. In comparison of the control meat samples at 6th and 12th days of storage, which were microbiologically not acceptable for consumption, the rate of reduced APC at 6th and 12th days by OmEO treatment was 2.5 Log 10 CFU/g, which was found to be greater in the previously reported data.^17,45,47,49,50^

It has been well demonstrated in a review of Domínguez et al.^28^ that a direct contact or headspace release of EO in meat packaging caused reduced lipid oxidation during the storage period, and the level of inhibited lipid oxidation in meat samples with direct contact of EO was greater than the level in meat samples in the case of headspace release of EO. Direct mixing of the meat with EO resulted in largely lowered lipid oxidation^8,20,22,28,48,51^ but caused a lowered general acceptability by the consumer.^22^ Our findings also supported these results in terms of reduced TBA and peroxide values by EO despite the fact that there is no research finding with active packaging (headspace) using EO. The rate of reduction in lipid oxidation is more likely depending upon the amount of EO used in active packaging, which should not exceed the level adversely affecting the odor of the product.^28^ In our study, the odor of meat is not adversely affected until the 12th day of storage by the OmEO amount used to coat the paper, and moreover, OmEO markedly lowered the peroxide value both at 6th and 12th days of storage. In general, the meat treated with EO in the form of active packaging has lowered lipid oxidation during cold storage.

In the light of our results and those reported previously,^8,11,29,30,48^ it is evident that carbohydrate-based films and papers with OmEO or other plant EO have greater antimicrobial and antioxidant effects and are found effective for prolonging the product shelf-life. These effects are likely due to an increased antibacterial and antioxidant property of packaging materials containing carbohydrates/polysaccharides loaded with EO.^52−54^

**Figure 4:**
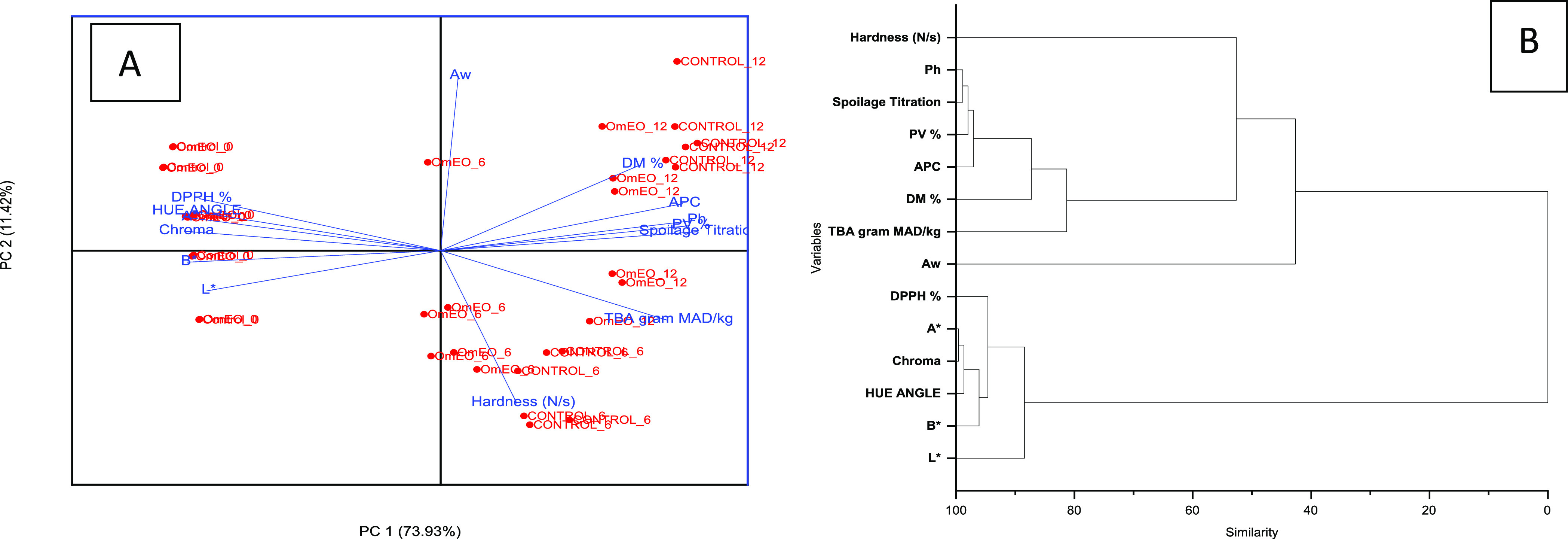
(A) Biplot of PCA analyses indicating a discrimination between the minced meat packed with the coated and noncoated papers stored for 0, 6, and 12 days. The biplot demonstrates the most influencing parameters in the discriminated meat samples. (B) The dendogram shows the degree of similarity between the parameters (variables).

A PCA analysis was carried out with all experimental parameters. A biplot indicated that the fresh meat (0th day) of both treatment groups was distinctively separated from the cold-stored meat samples at 6th and 12th days (Figure 4A). Moreover, there is a clear difference between the meat of the OmEO-coated group and that of the control group at 6th and 12th days of storage, while the meat of the control group at the 12th day of storage was markedly separated into a far coordinate of two components in the score plot. These results demonstrated an overall view of the minced beef influenced by an interaction of coating paper by storage period. In the detailed examination of the biplot, it can be noted that the color parameters and DPPH scavenging activity are the most influencing parameters of the fresh minced meat at the 0th day of storage, whereas PV, pH, HCl titrate value, and APC together with DM, TBA, and hardness are the most influential factors needed to evaluate the meat stored for a longer period (Figure 4A,B). Using a cluster analysis based on correlation coefficient values, a dendogram (Figure 4B) showed high similarities within each of the following two clusters: cluster 1 is “hardness-pH-HCl titrate-PV-APC-DM-TBA-*A*_w_” and cluster 2 is “Chroma-a*-Hue angle-DPPH-L*-b*”. The parameters within each cluster had high correlation coefficients and were used to well discriminate the degree of physical, chemical, and microbiological meat spoilage and deterioration. These results indicated that DPPH scavenging activity and physical parameters are important discriminations to identify the freshness and acceptability of meat products. On the other hand, chemical and microbiological parameters (pH, HCl titrate, peroxide value, TBA, APC, and DM) are excellent indicators to define the degree of microbial spoilage and lipid oxidation in cold-stored meat products for long periods.

## Conclusions

In conclusion, the production of papers coated with OmEO-cationic starch required a simplified and low-cost technology. OmEO was immobilized and characterized on the paper by SEM and FTIR analysis. In comparison to the meat packed with non-OmEO-coated paper, active packaging of minced meat with OmEO led to marked reduction in microbial growth by up to the 12th day of cold storage and maintained meat color parameters and sensory quality at the consumer acceptability level. Moreover, OmEO-coated paper led to a 7% decreased HCl titrate, 22% decreased peroxide value, 10% decreased TBA, and 40% increased DPPH scavenging activity. Having considered a general consumer acceptability, the present results suggested that active packaging with OmEO can be used to prolong minced beef up to at least 6 days of cold storage.

## References

[ref1] da Silva B. D.; Bernardes P. C.; Pinheiro P. F.; Fantuzzi E.; Roberto C. D. Chemical composition, extraction sources and action mechanisms of essential oils: Natural preservative and limitations of use in meat products. Meat Science. 2021, 176, 108463 10.1016/j.meatsci.2021.108463. 33640647

[ref2] Chivandi E.; Dangarembizi R.; Nyakudya T. T.; Erlwanger K. H.; Use of Essential Oils as a Preservative of Meat. In Essential Oils in Food Preservation, Flavor and Safety.; Preedy V. R., Ed.; Academic Press, 2016; pp. 85–91.

[ref3] Rodriguez A.; Nerín C.; Batlle R. New cinnamon-based active paper packaging against Rhizopusstolonifer food spoilage. J. Agric. Food Chem. 2008, 56, 6364–6369. 10.1021/jf800699q. 18627161

[ref4] Güvensen N. C.; Keskin D. Statistical determination of in-vitro antimicrobial effects of extracts of marjoram (*Origanum majorana* L.) from Muğla, Turkey. Int. J. Agric. Environ. Food Sci. 2021, 5, 388–394. 10.31015/jaefs.2021.3.19.

[ref5] Busatta C.; Vidal R. S.; Popiolski A. S.; Mossi A. J.; Dariva C.; Rodrigues M. R. A.; Corazza F. C.; Corazza M. L.; Vladimir O. J.; Cansian R. L. Application of *Origanum majorana* L. essential oil as an antimicrobial agent in sausage. Food Microbio. 2008, 25, 207–211. 10.1016/j.fm.2007.07.003. 17993397

[ref6] Van Haute S.; Raes K.; Van der Meeren P.; Sampers I. The effect of cinnamon, oregano and thyme essential oils in marinade on the microbial shelf life of fish and meat products. Food Control. 2016, 68, 30–39. 10.1016/j.foodcont.2016.03.025.

[ref7] Amariei S.; Poroch-Seriţan M.; Gutt G.; Oroian M.; Ciornei E. Rosemary, Thyme and Oregano Essential Oils Influence on Physicochemical Properties and Microbiological Stability of Minced Meat. J. Microbiol., Biotechnol. Food Sci. 2016, 1, 670–676. 10.15414/jmbfs.2016.6.1.670-676.

[ref8] Saricaoglu F. T.; Turhan S. Performance of mechanically deboned chicken meat protein coatings containing thyme or clove essential oil for storage quality improvement of beef sucuks. Meat Sci. 2019, 158, 107912 10.1016/j.meatsci.2019.107912. 31421517

[ref9] Guerrero A.; Ferrero S.; Barahona M.; Boito B.; Lisbinski E.; Maggi F.; Sañudo C. Effects of active edible coating based on thyme and garlic essential oils on lamb meat shelf life after long-term frozen storage. J. Sci. Food Agric. 2020, 100, 656–664. 10.1002/jsfa.10061. 31577841

[ref10] Almasi L.; Radi M.; Amiri S.; McClements D. J. Fabrication and characterization of antimicrobial biopolymer films containing essential oil-loaded microemulsions or nanoemulsions. LWT Food Sci. Technol. 2021, 117, 106733 10.1016/j.foodhyd.2021.106733.

[ref11] Yu H.; Li Y.; Lu S.; Wang Q.; Dong J. Effect and mechanism of thyme microcapsules on histamine production by Morganella morganii MN483274 during the processing of smoked horse meat sausage. Food Control. 2021, 121, 107615 10.1016/j.foodcont.2020.107615.

[ref12] Chaichi M.; Mohammadi A.; Badii F.; Hashemi M. Triple synergistic essential oils prevent pathogenic and spoilage bacteria growth in the refrigerated chicken breast meat. *Biocatalysis and Agricultural*. Biotechnology. 2021, 32, 101926 10.1016/j.bcab.2021.101926.

[ref13] García-Díez J.; Alheiro J.; Pinto A. L.; Soares L.; Falco V.; Fraqueza M. J.; Patarata L. Behaviour of food-borne pathogens on dry cured sausage manufactured with herbs and spices essential oils and their sensorial acceptability. Food Control. 2016, 59, 262–270. 10.1016/j.foodcont.2015.05.027.

[ref14] Min T.; Sun X.; Yuan Z.; Zhou L.; Jiao X.; Zha J.; Zhu Z.; Wen Y. Novel antimicrobial packaging film based on porous poly (lactic acid) nanofiber and polymeric coating for humidity-controlled release of thyme essential oil. LWT 2021, 135, 110034 10.1016/j.lwt.2020.110034.

[ref15] Almasi L.; Azizi S.; Amjadi S. Development and characterization of pectin films activated by nanoemulsion and Pickering emulsion stabilized marjoram (*Origanum majorana* L.) essential oil. Food Hydrocolloids 2020, 99, 105338 10.1016/j.foodhyd.2019.105338.

[ref16] Chaudhari A. K.; Singh V. K.; Das S.; Prasad J.; Dwivedy A. K.; Dubey N. K. Improvement of in vitro and in situ antifungal, AFB1 inhibitory and antioxidant activity of *Origanum majorana* L. essential oil through nanoemulsion and recommending as novel food preservative. Food Chem. Toxicol. 2020, 143, 111536 10.1016/j.fct.2020.111536. 32640350

[ref17] Emiroglu Z. K.; Yemis G. P.; Coskun B. K.; Candogan K. Antimicrobial activity of soy edible films incorporated with thyme and oregano essential oils on fresh ground beef patties. Meat Sci. 2010, 86, 283–288. 10.1016/j.meatsci.2010.04.016. 20580990

[ref18] Ozturk I. Antifungal Activity of Propolis, Thyme Essential Oil and Hydrosol on Natural Mycobiota of Sucuk, a Turkish Fermented Sausage: Monitoring of Their Effects on Microbiological, Color and Aroma Properties. J. Food Process. Preserv. 2015, 39, 1148–1158. 10.1111/jfpp.12329.

[ref19] Yemiş G. P.; Candoğan K. Antibacterial activity of soy edible coatings incorporated with thyme and oregano essential oils on beef against pathogenic bacteria. Food Sci. Biotechnol. 2017, 26, 1113–1121. 10.1007/s10068-017-0136-9. 30263643 PMC6049546

[ref20] Soncu E. D.; Özdemir N.; Arslan B.; Küçükkaya S.; Soyer A. Contribution of surface application of chitosan-thyme and chitosan-rosemary essential oils to the volatile composition, microbial profile, and physicochemical and sensory quality of dry-fermented sausages during storage. Meat Sci. 2020, 166, 108127 10.1016/j.meatsci.2020.108127. 32247159

[ref21] Ardjoum N.; Chibani N.; Shankar S.; Fadhel Y. B.; Djidjelli H.; Lacroix H. Development of antimicrobial films based on poly(lactic acid) incorporated with Thymus vulgaris essential oil and ethanolic extract of Mediterranean propolis. Int. J. Biol. Macromol. 2021, 185, 535–542. 10.1016/j.ijbiomac.2021.06.194. 34216656

[ref22] Pelaes Vital A. C.; Guerrero A.; Guarnido P.; Cordeiro Severino I.; Olleta J. L.; Blasco M.; Nunes do Prado I.; Maggi F.; Campo M. D. M. Effect of Active-Edible Coating and Essential Oils on Lamb Patties Oxidation during Display. Foods 2021, 10, 263 10.3390/foods10020263. 33513927 PMC7911211

[ref23] Vital A. C. P.; Guerrero A.; Monteschio J. D. O.; Valero M. V.; Carvalho C. B.; de Abreu Filho B. A.; Madrona G. S.; Prado I. N. Effect of Edible and Active Coating (with Rosemary and Oregano Essential Oils) on Beef Characteristics and Consumer Acceptability. PLoS One 2016, 11, e0160535 10.1371/journal.pone.0160535. 27504957 PMC4978481

[ref24] Granato D.; Nunes D. S.; Barba F. J. An integrated strategy between food chemistry, biology, nutrition, pharmacology, and statistics in the development of functional foods: A proposal. Trends Food Sci. Technol. 2017, 62, 13–22. 10.1016/j.tifs.2016.12.010.

[ref25] Lorenzo J.M.; Domínguez R.; Carballo J.; Control of lipid oxidation in muscle food by active packaging technology. In Natural antioxidants. applications in foods of animal origin.; Banerjee R.; Verma A.K.; Siddiqui M.W.; Ed.; Apple Academic Press, 2017, pp.343–382.

[ref26] Poojary M. M.; Putnik P.; Bursać Kovačević D.; Barba F. J.; Lorenzo J. M.; Dias D. A.; Shpigelman A. Stability and extraction of bioactive sulfur compounds from Allium genus processed by traditional and innovative technologies. J. Food Compos. Anal. 2017, 61, 28–39. 10.1016/j.jfca.2017.04.007.

[ref27] Vinceković M.; Viskić M.; Jurić S.; Giacometti J.; Kovačević D. B.; Putnik P.; Jambrak A. R. Innovative technologies for encapsulation of Mediterranean plants extracts. Trends Food Sci. Technol. 2017, 69, 1–12. 10.1016/j.tifs.2017.08.001.

[ref28] Domínguez R.; Barba F. J.; Gómez B.; Putnik P.; Kovačević D. B.; Pateiro M.; Santos E. M.; Lorenzo J. M. Active packaging films with natural antioxidants to be used in meat industry: A review. Food Res. Int. 2018, 113, 93–101. 10.1016/j.foodres.2018.06.073. 30195551

[ref29] Cai C.; Ma R.; Duan M.; Deng Y.; Liu T.; Lu D. Effect of starch film containing thyme essential oil microcapsules on physicochemical activity of mango. LWT 2020, 131, 109700 10.1016/j.lwt.2020.109700.

[ref30] Cui H.; Lu J.; Li C.; Lin L. Fabrication of phospholipid nanofibers containing eugenol@cationic starch nanoparticles against Bacillus cereus in beef. LWT 2021, 144, 111262 10.1016/j.lwt.2021.111262.

[ref31] ISO 11024-1.; Essential oils - General guidance on chromatographic profiles - Part 1: Preparation of chromatographic profiles for presentation in standards; International Organization for Standardization: Geneva 1998.

[ref32] ISO 11024-2. Essential oils - General guidance on chromatographic profiles - Part 2: Utilization of chromatographic profiles of samples of essential oils; International Organization for Standardization: Geneva 1998.

[ref33] Dal A. E. B.; Hubbe M. A.; Pal L.; Gule M. E. Crude Wood Rosin and Its Derivatives as Hydrophobic Surface Treatment Additives for Paper and Packaging. ACS Omega 2020, 5, 31559–31566. 10.1021/acsomega.0c03610. 33344808 PMC7745214

[ref34] Anonymous; Türk Gıda Kodeksi Et ve Et Ürünleri Tebliği (Tebliğ No: 2012/74). Turkish Food. Meat and Meat Products Directive. Accessed 20.08.2021, https://resmigazete.gov.tr/eskiler/2012/12/20121205-12.htm.

[ref35] Nizamlioglu N. M.; The effects of roasting and storage conditions on some physical, chemical and sensory properties of almond kernel. Pamukkale University, Graduate School of Natural and Applied Sciences, Department of Food Engineering. PhD Thesis, Denizli, 2015.

[ref36] Koniecko E. S.; Handbook of meat analysis; Avery Publication Group Inc. 1985.

[ref37] Martins J. T.; Cerqueira M. A.; Vicente A. A. Influence of α-tocopherol on physicochemical properties of chitosan-based films. Food Hydrocolloids 2012, 27, 220–227. 10.1016/j.foodhyd.2011.06.011.

[ref38] ICMSF.; Microorganism in Foods; Samples for Microbiological Analysis: Principles and Specific Applications, Recommendation of the International Commission on Microbiological Specification for Foods. Association of Microbiological Societies University of Toronto Press. 1985.

[ref39] Chang Y.; Mclandsborough L.; McClements D. J. Fabrication, stability and efficacy of dual-component antimicrobial nanoemulsions: Essential oil (thyme oil) and cationic surfactant (lauric arginate). Food Chem. 2015, 172, 298–304. 10.1016/j.foodchem.2014.09.081. 25442557

[ref40] Nisar T.; Wang Z. C.; Yang X.; Tian Y.; Iqbal M.; Guo Y. Characterization of citrus pectin films integrated with clove bud essential oil: Physical, thermal, barrier, antioxidant and antibacterial properties. Int. J. Biol. Macromol. 2018, 106, 670–680. 10.1016/j.ijbiomac.2017.08.068. 28818729

[ref41] Agatonovic-Kustrin S.; Ristivojevic P.; Gegechkori V.; Litvinova T. M.; Morton W. D. Essential Oil Quality and Purity Evaluation via FT-IR Spectroscopy and Pattern Recognition Techniques. Appl. Sci. 2020, 10, 7294 10.3390/app10207294.

[ref42] Moghimi R.; Aliahmadi A.; Rafati H. Antibacterial hydroxypropyl methyl cellulose edible films containing nanoemulsions of Thymus daenensis essential oil for food packaging. Carbohydr. Polym. 2017, 175, 241–248. 10.1016/j.carbpol.2017.07.086. 28917862

[ref43] Celebioglu A.; Yildiz Z. I.; Uyar T. Thymol/cyclodextrin inclusion complex nanofibrous webs: Enhanced water solubility, high thermal stability and antioxidant property of thymol. Food Res. Int. 2018, 106, 280–290. 10.1016/j.foodres.2017.12.062. 29579928

[ref44] Yancey J. W. S.; Kropf D. H. Instrumental reflectance values of fresh pork are dependent on aperture size. Meat Sci. 2008, 79, 734–739. 10.1016/j.meatsci.2007.11.006. 22063037

[ref45] Zengin H.; Baysal H. Antioxidant and Antimicrobial Activities of Thyme and Clove Essential Oils and Application in Minced Beef. J. Food Process. Preserv. 2015, 39, 1261–1271. 10.1111/jfpp.12344.

[ref46] Hussain Z.; Li X.; Zhang D.; Hou C.; Ijaz M.; Bai Y.; Xiao X.; Zheng X. Influence of adding cinnamon bark oil on meat quality of ground lamb during storage at 4°C. Meat Sci. 2021, 171, 108269 10.1016/j.meatsci.2020.108269. 32768894

[ref47] Solomakos N.; Govaris A.; Koidis P.; Botsoglou N. The antimicrobial effect of thyme essential oil, nisin, and their combination against Listeria monocytogenes in minced beef during refrigerated storage. Food Microbiol. 2008, 25, 120–127. 10.1016/j.fm.2007.07.002. 17993385

[ref48] Cai L.; Leng L.; Cao A.; Cheng X.; Li J. The effect of chitosan essential oils complex coating on physicochemical, microbiological, and quality change of grass carp (Ctenopharyhgodon idella) fillets. J. Food Saf. 2018, 38, 12399 10.1111/jfs.12399.

[ref49] Abdollahzadeh E.; Rezaei M.; Hosseini H. Antibacterial activity of plant essential oils and extracts: The role of thyme essential oil, nisin, and their combination to control Listeria monocytogenes inoculated in minced fish meat. Food Control. 2014, 35, 177–183. 10.1016/j.foodcont.2013.07.004.

[ref50] Agrimonti C.; White J. C.; Tonetti S.; Marmiroli N. Antimicrobial activity of cellulosic pads amended with emulsions of essential oils of oregano, thyme and cinnamon against microorganisms in minced beef meat. Int. J. Food Microbiol. 2019, 305, 108246 10.1016/j.ijfoodmicro.2019.108246. 31238193

[ref51] Can Ö. P.; Kaşıkçı G. Biberiye ve kekik yağı ilavesinin marine edilmiş gökkuşağı alabalıklarının (oncorhynchus mykiss walbaum 2018) buzdolabında depolanması üzerine etkisi. Turk. J. Agric.- Food Sci. Technol. 2018, 6, 1701–1707. 10.24925/turjaf.v6i12.1701-1707.1683.

[ref52] Fasihi H.; Noshirvani N.; Hashemi M.; Fazilati M.; Salavati H.; Coma V. Antioxidant and antimicrobial properties of carbohydrate-based films enriched with cinnamon essential oil by Pickering emulsion method. Food Packag. Shelf life. 2019, 19, 147–154. 10.1016/j.fpsl.2018.12.007.

[ref53] Lian H.; Shi J.; Zhang X.; Peng Y. Effect of the added polysaccharide on the release of thyme essential oil and structure properties of chitosan based film. Food Packag. Shelf Life. 2020, 23, 100467 10.1016/j.fpsl.2020.100467.

[ref54] Liu Z.; Lin Shen R.; Zhang R.; Liu L.; Yang X. Konjac glucomannan-based edible films loaded with thyme essential oil: Physical properties and antioxidant-antibacterial activities. Food Packag. Shelf Life. 2021, 29, 100700 10.1016/j.fpsl.2021.100700.

